# Towards Tangible Vision for the Visually Impaired through 2D Multiarray Braille Display

**DOI:** 10.3390/s19235319

**Published:** 2019-12-03

**Authors:** Seondae Kim, Yeongil Ryu, Jinsoo Cho, Eun-Seok Ryu

**Affiliations:** 1 Department of Computer Education, Sungkyunkwan University, Seoul 03063, Korea; ele7004@skku.edu; 2 Department of Computer Engineering, Gachon University, Seongnam-si, Gyeonggi-do 13120, Korea; yiryu@gc.gachon.ac.kr

**Keywords:** haptic telepresence, braille device, braille application, visually impaired people

## Abstract

This paper presents two methodologies for delivering multimedia content to visually impaired people with the use of a haptic device and braille display. Based on our previous research, the research using Kinect v2 and haptic device with 2D+ (RGB frame with depth) data has the limitations of slower operational speed while reconstructing object details. Thus, this study focuses on the development of 2D multiarray braille display using an electronic book translator application because of its accuracy and high speed. This approach provides mobility and uses 2D multiarray braille display to represent media content contour more efficiently. In conclusion, this study achieves the representation of considerably massive text content compared to previous 1D braille displays. Besides, it also represents illustrations and figures to braille displays through quantization and binarization.

## 1. Introduction

According to recent statistical research, the number of people who are visually impaired is steadily increasing. It categorized 36,000,000 people as legally blind, approximately 217,000,000 people had moderate to severe visual impairment, and 188,000,000 had mild vision impairment. Generally, people who have visual impairments have a decreased ability to see to a degree that causes problems not fixable by usual means, such as glasses. Moreover, as the population and the age increase, the number of people who lose their sight increases proportionally. The United States Census Bureau stated that the population of the United States is increasing every year [1] and as it grows older, the number of diseases affecting vision is also increasing [2]. Table 1 indicates the correlation between age and prevalence of vision disorders [3].

To solve the practical difficulties of visually impaired people, several researchers have studied smart canes, smart cameras with various sensors, braille displays, etc. In general, smart canes consist of electronic navigation aids attached to canes for visually impaired people for detecting obstacles and aiding in practical road navigation. In addition, many studies that focused on canes have applied electro-tactile devices, vibration sensors, ultrasonic sensors, and auditory feedback for assistive devices [4]. The methods using cameras contribute to vision sensing, text reading, guidance, navigation, and obstacle detection. In particular, Microsoft Kinect is significantly useful for obtaining a 3D depth image or video for detecting 3D obstacles in the real world. Kinect has infrared (IR) sensors and an RGB camera. To telepresent 3D shape information, haptic devices are useful for depicting 3D objects. For a considerable amount of time, braille displays or reading materials have been studied for interpreting literal content (e.g., news, books, documents) and convert it to braille.

This research attempts to express a tactual vision through a haptic device to depict 3D objects. Further, this paper shows braille applications for a next-generation braille display that has 2D multiarray braille cells to describe media content (e.g., text content, photographs, paintings) with audio feedback for multimedia information delivery. The contributions of this study toward sharing multimedia content with visually impaired people are as follows:

- Firstly, this study presents a haptic telepresence system that the visually impaired people can use to feel the shape of a remote object in real time. The server captures a 3D object, encodes a depth video using high efficiency video coding (HEVC)(H.265/MPEG-H) and sends it to the client. The 2D+depth video is originally reconstructed in 30 frames per second (fps) but the visually impaired people do not need such a high frame rate. Thus, the system downsamples the frame rate according to human feedback; the hpatic device has the button to let the system know the user finished exploring the frame. It can express a 3D object to visually impaired people. However, the authors realized the limitations of this methodology; consequently, the focus of this study was turned to the research on a next-generation braille display. The limitations of haptic telepresence will be discussed in Section 6.
- This study also presents the 2D multiarray braille display. For sharing multimedia content, it presents a braille electronic book (eBook) reader application that can share a large amount of text, figures, and audio content. In the research steps, there are a few types of tethered 2D multiarray braille display devices; they are developed to simulate the proposed software solution before developing a hardware (HW) 2D braille device. The solution in HW device is that does not need the tethered system.

In this study, only the eBook reader application is considered. The authors hope that this research on the eBook-based braille display will help in designing other applications based on the multiarray braille display. This method also has its strengths and weaknesses.

This paper consists of multiple sections. Section 2 introduces related work with varying methods to help visually impaired people. There are related studies that present other viable support mechanisms for visually impaired people, such as tangible devices, alternative visions, and auditory feedback. Subsequently, both the haptic telepresence system and the eBook reader application based on the braille display interface are specifically discussed in Section 3 and Section 4. In Section 5, the implementation and experimental results are presented. This study discusses the limitations and current challenges in Section 6. Finally, Section 7, concludes the contribution of this paper.

## 2. Related Work

**Figure 1:**
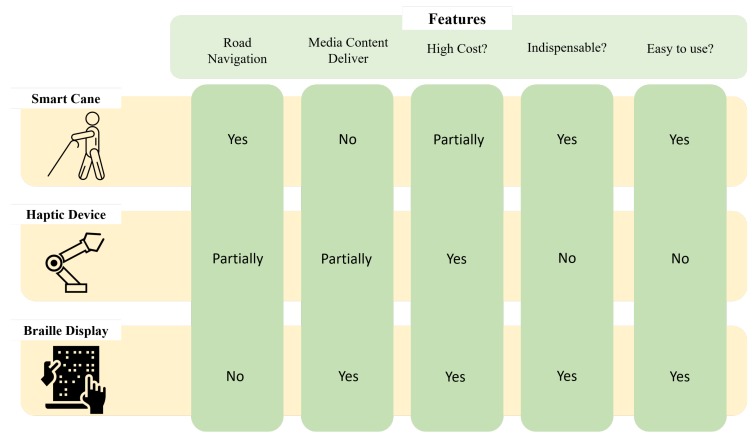
Comparison according to the features of assistive devices for the visually impaired.

This section introduces various assistive devices for the visually impaired. There are several helpful devices using ultrasonic sensors, vision, and the sense of touch. Figure 1 illustrates the criteria considered by the visually impaired people while purchasing assistive devices. Each device has different objectives practically. For example, most of the visually challenged people can use a smart cane to recognize certain obstacles. It is a useful and an indispensable item when they are walking. Conversely, haptic devices or braille displays are not indispensable products. The necessity is different for each person corresponding to their jobs. Comparatively, the braille display is more useful than a haptic device. It provides media information through the internet and it is also easy to use.

### 2.1. Smart Canes Using Various Sensors

Until now, one of the most popular studies concerning people with visual impairments was regarding the smart cane. Smart canes have been studied in various ways. Laser sensors, ultrasonic sensors, and cameras are usually used to upgrade canes and provide more accurate guide services. One of the earliest studies on smart canes used a laser sensor. Bolgiano et al. [5] are considered the pioneers on research regarding smart canes. They developed a smart cane with relatively high energy conversion efficiency, because the smart canes operated with a lower battery capacity previously. The technology was coupled with the time of use to build a better pulse system that achieved a significantly small duty cycle of approximately one to one million.

**Figure 2:**
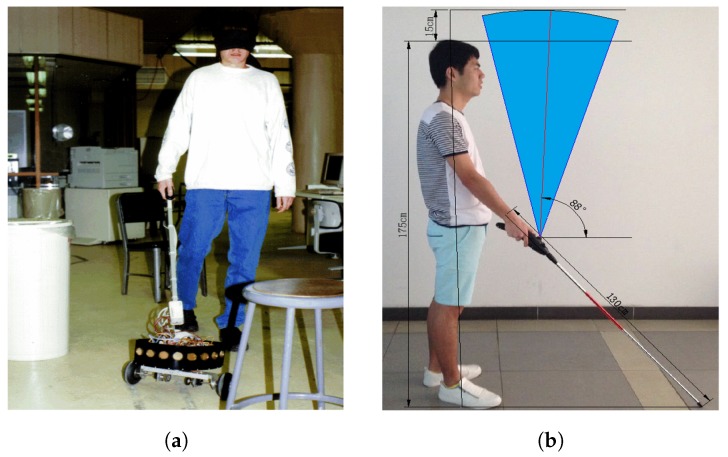
(**a**) Blindfolded graduate student. This student walks through an obstacle course using the guide cane (**b**) Detection range of the sensor. It covers a range above and in front of the user [7].

The ultrasonic sensor was beneficially utilized in smart canes, which has resulted in several studies using ultrasonic sensors in smart canes. Borenstein et al. [6] developed a smart cane with ultrasonic sensors, as shown in Figure 2a, which could detect at an angle of $180^{\circ }$ horizontally in the forward direction; thus, it was possible to distinguish between walls on the left and right of the person wielding the cane from objects $120^{\circ }$ vertically in the forward direction. The obstacle recognition method was developed using ultrasonic waves; consequently, rapid processing was possible and feedback on obstacles was provided in real time to match the speed at which the human walked. Yi et al. [7] developed a lighter and more convenient smart cane. Although this cane demonstrates certain blind spots, such as locations just beside a user, it is easy to carry and solves the limitation in determining obstacles above the height of the user. A study conducted by Wahab et al. [8] used ultrasonic sensors, a buzzer, a vibrator, and a fuzzy controller to customize the smart cane. This cane can be easily folded and provides feedback to users when obstacles are present through the vibrator and buzzer. It also has a water sensor to recognize more than 0.5 cm of water, which can warn users.

### 2.2. Communication of 2D or 3D Objects Using Haptic Device or Depth Camera

**Figure 3:**
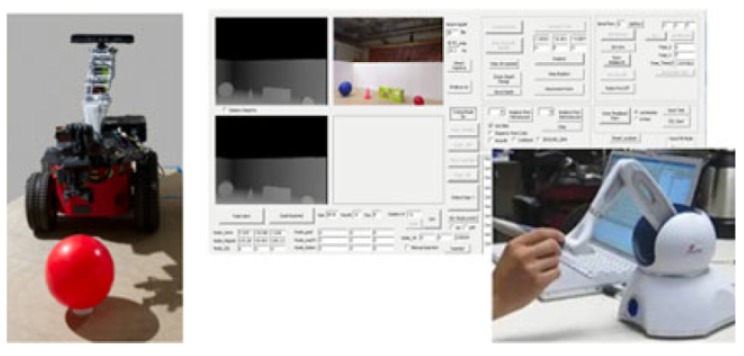
Telepresence robot with multimodal interface for visually impaired people [9].

There are methods that use haptic telepresence devices, which can express tactual perception using 2D+depth data. Park et al. [9,10,11,12,13], who collaborated with our team, developed the robot system to remotely observe the visual attractions and evade obstacles at art galleries or museums. Figure 3 illustrates a telepresence robot and a haptic interaction system. Previously, Park at al. studied remote robots that provide feedback to users and haptic devices that can be used for exploration. They focused on the following: (i) implementation of depth video projection system; (ii) development of a video processing method; and (iii) enhancing 2D+ depth map quality.

**Figure 4:**
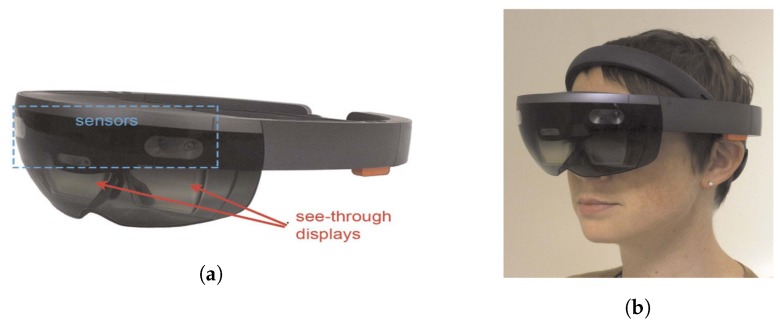
HoloLens hardware (**a**) HoloLens has binocular displays (red arrows), sensors (blue dashed box), and an onboard operating system (**b**) Users wear HoloLens by tightening an adjustable band around the head and position the screen in front of their eyes [16].

There are several studies using depth cameras. Hicks et al. [14] studied a link between depth cameras and a head-mounted display (HMD) which enabled people with low vision, i.e., not complete visual loss, to detect obstacles. They studied the images captured using a depth camera, identified the obstacles, and communicated the presence of obstacles using light-emitting diodes (LEDs). A low-resolution display illustrated the distance to nearby objects on a scale of brightness. Hong et al. [15] identified obstacles recognized by the cameras installed in car to enable visually impaired people to drive. Tactile actuators are installed on the handle and gloves to indicate the location of obstacles identified through video recognition. Kinateder et al. [16] avoided unnecessary equipment by replacing only the eyeglasses with augmented reality (AR) glasses for the visually impaired, as shown in Figure 4 for the visually imparied. The HoloLens (Microsoft) is a head-mounted AR system that can display 3D virtual surfaces within the physical environment. The major limitation of the HoloLens system is that it updates distance information at only up to 1 Hz; consequently, it may be difficult to perceive fast-moving objects. The authors expect that the lag and limited range of the mapping, which are clear limitations of the device, will improve with the next generations of HMDs.

### 2.3. Information Transfer Using Braille Device and Assistive Application

This topic has been studied more than other areas so far; moreover, it is a significantly effective method for visually impaired people to obtain useful information from books. Oliveira et al. [17] presented a text-entry method based on the braille alphabet. Further, this method avoids multi-touch gestures in favor of a simple single-finger interaction, featuring fewer and larger targets. Velázquez et al. [18] studied a computer-based system that automatically translates any eBook into braille. This application is called TactoBook, which displays the contents of eBooks converted into braille and can be transferred to prototype devices through a universal serial bus port. While there are certain inconveniences while transferring data from eBooks to a braille display through a separate storage device, it has simple interfaces for users and is easy to carry. In addition, Goncu and Marriott [19] produced an eBook image creator model based on iBooks (an iPad application), which utilizes graphic content for people with low vision.

**Figure 5:**
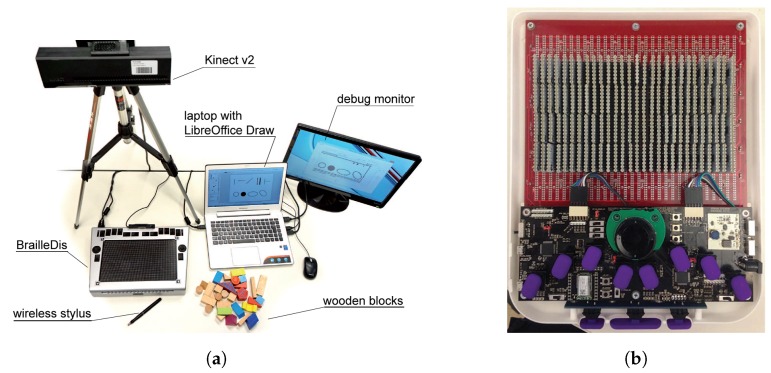
Related braille display works. (**a**): The drawing workstation with laptop computer, monitor, BrailleDis 6240 tactile pin-matrix device, ToF 3D camera system with wooden blocks for capturing, and wireless digitizer stylus [20]. (**b**): Final prototype internal components. The LED display is on the top, with the micro-controllers and keyboard below [22].

Bornschein et al. [20,21] studied tactile graphics to create figures by using braille to introduce information effectively for visually impaired people. Further, several previous projects address the quantitative aspects, such as the support of sighted graphics producers, to accelerate the direct transformation. By capturing the depth map from the IR sensor of Kinect v2, the silhouette extracted from objects can be utilized to create graphical shapes. Figure 5a illustrates the 2D braille workstation of Bornschein et al. Thus, the visually impaired people could communicate with non-visually impaired people through graphical expressions. Byrd et al. [22] presented a conceptual braille display similar to the display used in this study, which is illustrated in Figure 5b. It has a 4×28 LED braille display, Perkins-style braille keyboard, scroll wheel, and navigation buttons. Moreover, it supports Bluetooth communication with a PC for remote information exchange. However, this study is focused on only braille hardware and does not consider the software, such as the operating system (OS) or applications that are run in the braille display.

Additionally, several other researchers conducted studies to help visually impaired people. Bae [23] conducted a study to better serve visually impaired people who want to read utilizing the digital accessible information system (DAISY), which is a eBook standard for handicapped people. Further, Kim et al. [24,25] implemented a DAISY viewer into the smartphone. The DAISY v2.02 and v3.0 users are supported using smartphones so that people with reading disabilities can easily read DAISY eBooks anywhere. Harty et al. [26] studied a DAISY mobile application to allow access to DAISY-standard eBooks using Android OS. It supports DAISY v2.02 partially. The present study examined the DAISY v2.02 reader application based on this research. Moreover, it is difficult to obtain detailed information regarding the Android OS-based players that fully support DAISY v3.0 eBooks. Mahule [27] studied a DAISY v3.0 application based on Android smartphones; however, it has not been completely developed. Thus, this study redesigned his research for implementation. It is described in detail in Section 4. Further, significant studies were conducted by other companies; however, no useful information was released because of their intellectual property rights. The next-generation braille display presented in this paper is also a commercial product. To set better research goals, this paper introduces the current commercially popular braille displays. We clarify that the presented information is based on the published information in their manuals and websites [28,29,30,31,32]. Table 2 lists a comparison of their products with features.

Finally, this study collaborated with the InE lab at Gachon University to develop the next-generation display. They have developed a braille OS and its additional applications. Their system was utilized in this study for developing a braille eBook reader for multimedia information delivery. Park et al. [33] developed a method for automatically translating the scanned images from print books into electronic braille books with reduction in translation time and cost required for producing braille books. In addition, when compared to the traditional methods of obtaining information by only using braille text, the aforementioned studies have attempted to accurately transfer 2D graphics, and photographic data. These studies have researched the conversion of 2D graphics to braille data display using multiarray braille simulated in smartphones. Similarly, this study has researched an eBook reader application for a 2D multiarray braille display.

## 3. 3D Haptic Telepresence System

### 3.1. Architecture of 3D Haptic Telepresence System

Previous studies [34,35,36,37] have continuously attempted to enable visually impaired people to access visual information. As discussed in Section 1, the number of people who have poor vision or have lost their eyesight is increasing worldwide every year. Further, it is difficult for them to obtain visual multimedia from information technology devices, such as PCs, smartphones, and televisions. Park, a co-researcher [9,10,11,12,13], developed methods for touching 3D shapes through depth capturing and its reconstruction. However, these methods are considerably slow for real-time haptic telepresence. Thus, this study focused on real-time 3D haptic telepresence with moderate quality. Section 3.1 introduces a haptic interaction system by capturing a depth map, which upgraded the processing speed. This section introduces the 3D haptic telepresence in real time using Kinect v2 and a haptic device.

**Figure 6:**
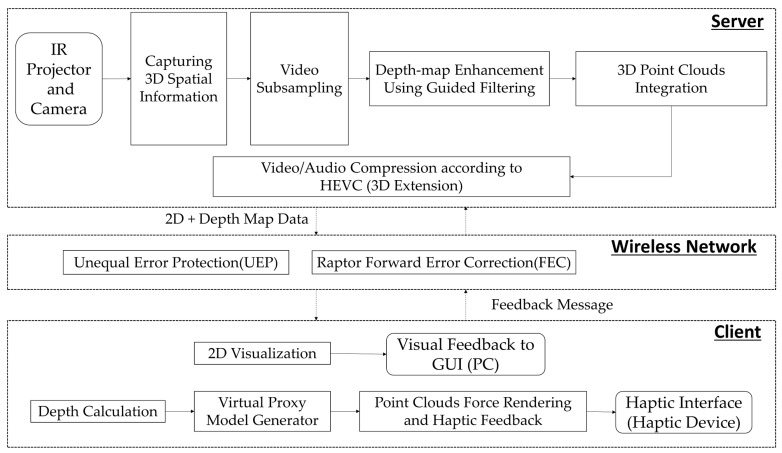
Telepresence system architecture for visually impaired people.

The studies conducted by Park [9,10,11,12,13] were focused on capturing 2D+ depth images and rendering using a haptic device. However, this study performs haptic rendering through a 2D+ depth video. The Kinect server can capture 3D spatial information of an object using an IR projector and a camera. The extracted information is processed and converted to a 3D point cloud form, which is then compressed through the latest compression technique, HEVC. The compressed image is delivered to a client through wireless communication, and the client receives the compressed depth map data. The received 2D image is expressed to a graphical user interface (GUI) in real time; further, the 3D point cloud information is expressed using a haptic device through a force rendering. Figure 6 shows architecture of 3D Haptic Telepresence System.

### 3.2. 3D Spatial Information Capture with Depth Map Using IR Projector and Camera

The 3D spatial information capture module was performed using the Microsoft Kinect software development kit (SDK) [38,39]. The Kinect SDK captures 2D+ depth images. The size of the 2D images are up to 1920×1080 (full high definition) pixels. The 3D depth map is up to 512×424 (close to standard definition) pixels. However, the haptic device that we studied has a limited resolution (320×240). This implies that the information projected to the 3D point cloud need be downsampled to a lower resolution. In addition, the 3D spatial information can be captured by using either stereo/multiview matching from RGB images or an IR projector and camera. Although the stereo/multiview matching can render more details than IR sensors, its computational complexity is also significantly higher than IR sensors. Thus, this capture module of the proposed system is implemented using IR sensors to provide real-time 2D+ depth video streaming service.

**Figure 7:**
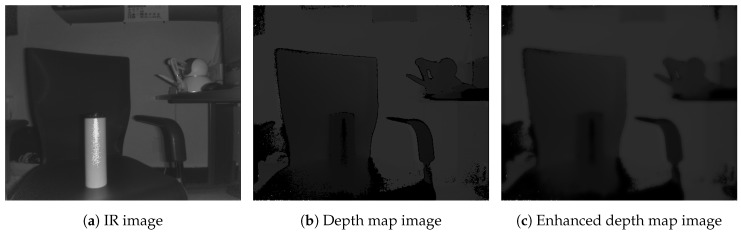
Depth map enhancement using guided filtering.

**Figure 8:**
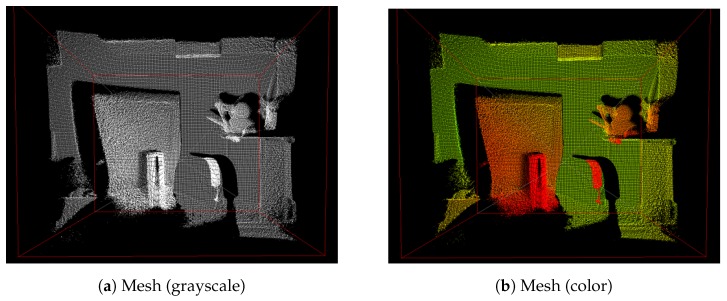
Mesh map(3D view) from Kinect v2 using Kinect Studio v2.0.

The depth map from IR sensors is generally noisy and not sufficiently accurate. It suffers from noise, such as jitter and unequalized edges, because of the distorted light wave fluctuation. Therefore, guided filtering, which is a linear translation-variant filtering process, is applied to the depth map improvement module. The guided filtering process smooths the image while preserving edges. Moreover, it is used to equalize the noises in the depth map [40,41]. Further, the guided filtering is lightweight, removes artifacts more efficiently, and provides better smoothing than bilateral filtering. Figure 7 shows sample results of the depth map enhancement using guided filtering in this work. As shown in Figure 7a, the original IR image was captured using Kinect v2; subsequently, Figure 7c illustrates the depth map image from the Kinect v2 SDK. Figure 7b shows the filtered data with guided filtering. From this guided filter image, point clouds can be constructed using the metadata of the depth map. This 3D metadata involves the coordinates (horizontal, vertical, and depth values) for the depth map projection. To make it easier to understand, in Figure 8, there are two grid maps (grayscale and color) obtained using Kinect v2 SDK. From the grid, objects that are closer (white or red) and farther (black or green) and the approximate shape of each object can be observed. This 3D metadata from the depth map can be used for creating a point cloud for haptic device projection though the OpenHaptics and CHAI3D library [42,43].

### 3.3. Real-Time Video Compression and Transmission to Haptic Device

To transmit multimedia combined with 3D spatial information in the form of a point cloud, 2D true-color image, and audio, compression is crucial in networks with limited bandwidth. The captured spatial data are too big to transfer to client in real time. To transmit spatial data efficiently, HEVC is used [44] to enable advanced compression. It reduces more than 50% of the bitrate when compared to existing video coding standards. Park et al. [9] studied haptic rendering with a 320×240 point cloud, which was transformed into a 3D grid map, translated into a disparity map for visualization, and calibrated for distance in case of scaling. In contrast, this research provides the depth map from IR sensors at approximately 30 fps. Although this research was directly scaled for transmission, more effective streaming of 3D images is possible using scalable HEVC [45,46].

Additionally, HEVC considers a 3D extension for depth map compression, which also includes multiview video compression. The 3D HEVC extension uses the view concept instead of the frame concept, because 2D frames are captured at a fixed angle, but 3D frames have multiple angles. Thus, 3D compression uses the view concept for interprediction and intraprediction, view synthesis, and dealing with the prediction unit, error correction technique, and motion compensation methodology, by aligning the motion vectors by view. The sequences on 2D and 3D HEVC are quite similar; however, there are additional techniques involved in the 3D HEVC, such as view synthesis prediction to reduce the inter-view redundancy and partition-based depth intra coding (e.g., depth modeling modes). This study processed the depth video/image view according to the feedback from the user by using an original 3D HEVC extension. When users want to view the next depth image (view), as shown in Figure 6, it appropriately encodes the next 2D+ depth view for the client [44,47,48,49].

This study assumes that a server system can be connected using a wireless network. For reliable delivery, the network module includes rate control, Raptor forward error correction, and unequal error protection to improve the quality of service. In particular, these techniques are very useful to protect data and provide robust data transmission over the wireless network [50,51]. In addition, they enable the optimization of the data packet size. The compressed data involve large buffer streams generated from a depth sensor. After the server completes the transmission of a clean 2D+ depth map data to the client using the transmission control protocol/internet protocol, the haptic device can depict the appearances of objects [43]. The server and client exchange their data, and data can be transferred steadily. Then, the client side proceeds to decompress video data.

### 3.4. Real-Time Haptic Interaction Using 2D+ Depth Video

**Figure 9:**
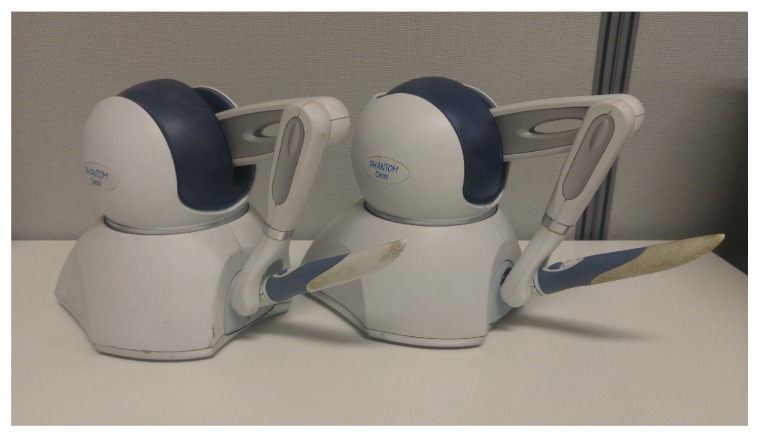
PHANTOM Omni haptic devices from SenAble Technologies. The company provided drivers and related software to utilize them.

To optimize haptic feedback, a haptic interaction module is implemented using the CHAI3D library and OpenHaptics [42,43]. CHAI3D provides several kinds of examples to utilize haptic devices. As shown in Figure 9, this haptic device has a haptic pencil on the arm. It describes the edges of object to users; moreover, it has a button for requesting the next depth frame. Furthermore, the OpenHaptics toolkit is a framework that supports low-level controls in the haptic device. This study has applied haptic projection examples and their 3D projection algorithms, such as manipulation of a haptic device, correcting boundaries, coordinate scaling. Applications also include 3D designed visualization and simulation. When the client receives the 2D+ depth video data, it is processed using a virtual proxy algorithm. The occupied and unoccupied points can be used to calculate and express the volume of captured objects with virtual proxy force. The force rendering that is used for depicting the objects is termed virtual proxy force. It is similar to a spring–damper model, as expressed by (1).

(1) $${\vec{F}}_{feedback}=k\left({\vec{P}}_{proxy}-{\vec{P}}_{probe}\right)+b\left({\vec{V}}_{proxy}-{\vec{V}}_{probe}\right)$$

Given the position vector of the proxy ${\vec{P}}_{proxy}$, the position vector of the probe ${\vec{P}}_{probe}$, and velocities of the proxy and the probe ${\vec{V}}_{proxy}$ and ${\vec{V}}_{probe}$, respectively, a virtual proxy force feedback ${\vec{F}}_{feedback}$ is composed of a penetration depth term (with a spring constant k) and damping term (with a damping constant b). ${\vec{P}}_{proxy}$ and ${\vec{P}}_{probe}$ are static values that represent the object shape and stiffness on the haptic device. The damping term is a dynamic vale that conveys the movement of the probe. This model is widely adopted in haptic rendering. When translating the depth map, data points consist of a virtual proxy model within neighboring 3D points. These points, called haptic interaction points, are used to calculate 3D surface estimation [9]. Furthermore, the feedback could be shown when the user wants to recognize the subsequent frames; generally, an input is provided using a button on the pencil of the haptic device. This real-time interaction allows the system to have robust data translation, i.e., generating visualization feedback or representing dynamic movements in real time.

## 4. Electronic Book (eBook) Reader Application for 2D Braille Display

### 4.1. Design of 2D Multiarray Braille Eisplay and Its Architecture of eBook Reader Application

**Figure 10:**
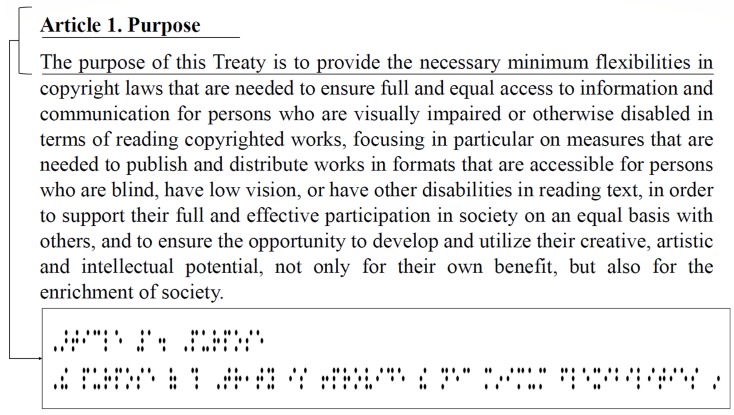
Quantitative comparison between text and braille content.

This section presents the design of the next-generation braille display device and its eBook reader application. Moreover, it explains the method used to convert the massive text to a braille display page, and the method used for braille figure conversion. So far, most of the mobile braille displays were composed of single-lined braille cells, which demonstrate the following disadvantages: (i) when the braille text shown in the braille cells is limited to a single line, it is inconvenient to read a book; (ii) images or videos are expressed with some of the text sentences; and (iii) an assistant of a visually impaired person must know the braille letters to sufficiently understand the multimedia content. The first problem occurs because braille cells are expressed in a single line; therefore, it is difficult to understand certain text (e.g., poetry, quotation) in a single reading. In addition, if there is a significant amount of text content, a certain button has to be pressed repeatedly to turn over the pages. For example, as shown in Figure 10, a large amount of text can be expressed simply for normal people whereas the number of braille cells for the same text is physically increased. Consequently, when visually impaired people read this text, there will be a difference in the reading level when compared to non-visually impaired people.

When visually impaired people choose a braille device, they generally consider mobility, Perkins-style keyboards, different numbers of cells (32 is the popular choice), a supportive system for the braille device, etc. However, the panning problem is the primary issue while obtaining media information. When a given text is too long to fit in the display screen, the user has to “pan” either to the left or the right to continue reading along the line. For example, while using a 20-cell braille display, to read a 72-character line, the reader will have to pan at least twice to read the entire text [52]. This study utilizes the braille OS designed by InE lab, which is based on a 2D multiarray braille display, because it partially solves the second problem. For the third problem, this study provides screen mirroring between visually impaired and non-visually impaired people.

**Figure 11:**
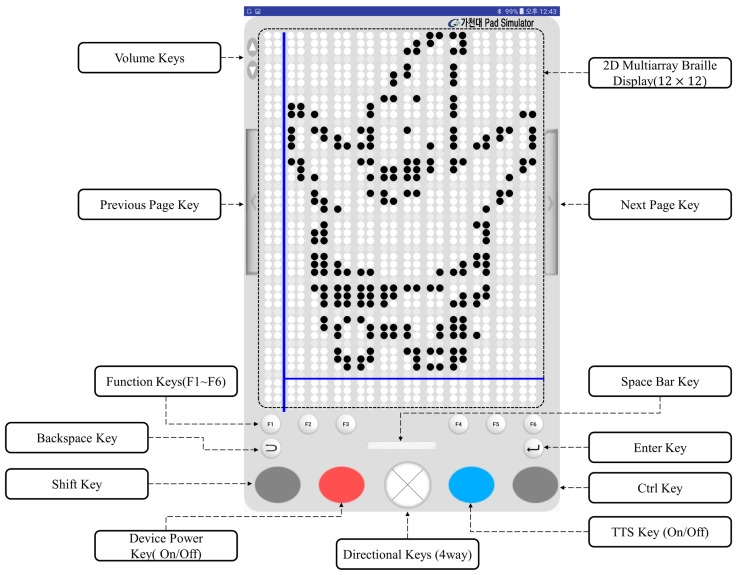
Design of braille pad simulator. There are keyboards and 2D multiarray braille display (12×12 braille cells). It is operated on a tablet PC.

In this study, the braille display is designed to solve the aforementioned problems. As shown in Figure 11, 12×12 braille cells are located on the braille pad. The volume keys located beside it can be used to increase or decrease the volume of audio or text-to-speech (TTS). Using a long click, the speed of the TTS can be increased or decreased. It has page keys for turning a page to the next or previous page, when reading the texts that have pages similar to an eBook. In addition, six function keys, the backspace key, enter key, space key, directional keys, shift key, control key, device power on/off key, and TTS key exist on the braille pad. These keys perform various roles, such as playing or pausing TTS/audio file and saving/fetching/deleting bookmarks.

### 4.2. Modules and Application on Braille OS

**Figure 12:**
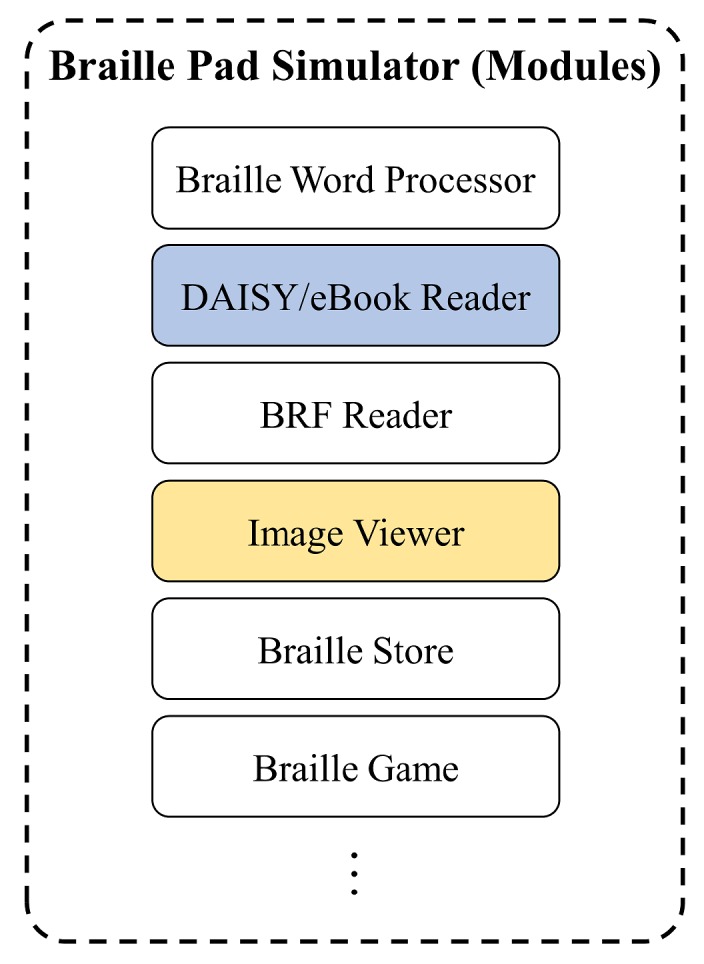
Representative modules on braille simulator. This study treats the digital accessible information system (DAISY)/electronic book (eBook) reader only. In addition, the image viewer is partially used to display braille figures.

**Figure 13:**
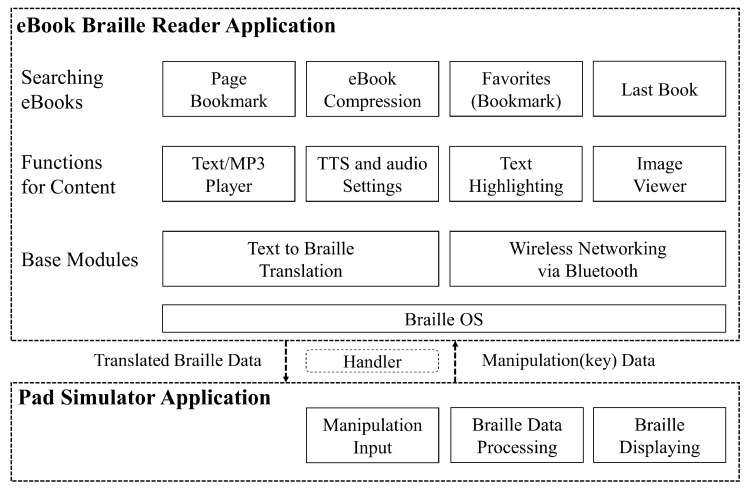
Proposed eBook braille reader application architecture for 2D multiarray braille display.

This braille device was developed by a joint research team. The braille OS, image viewer, and other applications were developed at the InE lab at Gachon University. As mentioned before, they had already developed a technology that can convert visual illustrations to tactual braille images. As illustrated in Figure 12, there are modules of the braille display simulator that can assist visually impaired people. This study conducted the research among the visually impaired people regarding the braille eBook reader application module. As shown in Figure 13, the visual content, such as text and image contents, are expressed through the tablet that has a multiarray braille display. The computation related to the expression of visual/audio content is primarily performed using a smartphone. The media content is tangibly expressed through the 2D multiarray braille display. Further, the video content is shown using a cover image, such as a thumbnail. Moreover, the mirroring between the braille display device (in this study, it was analyzed using the braille pad simulator application on the tablet) and a regular smartphone application can solve the aforementioned third problem. A smartphone can express its original language.

Figure 13 illustrates the structure of the eBook reader application studied in this research. This application is implemented based on DAISY v2.02 and v3.0 [53], which are specialized standards for disabled people. Moreover, the international electronic publication (EPUB) standards, i.e., EPUB 2.0 and 3.0 [54] are also available. Every book under this standard is implemented to be compatible with the braille OS. The books are compressed in different backgrounds; page management is also available for 2D multiarray braille display. Further, the content display, text highlight, audio player, TTS, the speaking rate, and the pitch of TTS can be controlled by users. In addition, the following functions are also provided in this braille eBook reader application: a function for searching a book stored in the storage of the smartphone, saving/opening a book that was read most recently, bookmark function, favorites function, book or bookmark deletion function, and book compression/decompression function for efficient use of storage space. All contents are translated to braille and sent to the braille tablet. The braille translation is performed using a braille translation module (engine) developed by Dot Inc., which is company that manufactures assistive devices. It can translate both English and Korean for the braille devices.

### 4.3. Wireless Mirroring between Braille Pad and Smartphone

The braille OS is installed on both the smartphone and pad simulator (tablet). Messages are produced on the eBook reader application on the smartphone; moreover, those messages are transmitted to the braille pad simulator. The messages for sharing media data are transmitted and received through a wireless network. The Android OS message object is used for this function, which is provided by default in the Android OS [55]. This object facilitates sending and receiving messages between devices by using a handler, and the key code used for a message is defined. The key code is transmitted from the braille pad simulator, which is used by visually impaired users. This study defined the data of the key with 16 bits.

**Figure 14:**
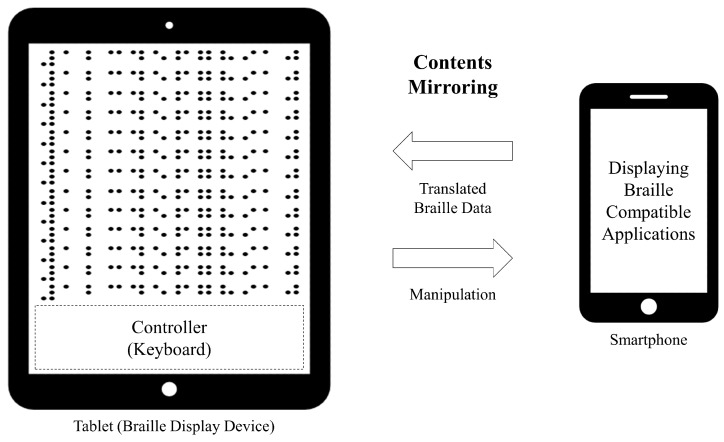
Screen mirroring between the smartphone and the tablet. This mirroring can share every activity on the braille operating system (OS). The smartphone displays original multimedia contents for non-visually impaired people. The tablet display translates multimedia content converted to braille for visually impaired people.

Moreover, the braille OS is a background application. When the on/off button is pressed on the pad, the Android activity of the smartphone [56] is automatically displayed. Subsequently, the visually impaired users can remotely control the smartphone with the tablet by using the braille pad. Then, the eBook application is activated, as shown in Figure 13. All the data of the eBook is sent to the smartphone at a rate of a byte per braille cell. This is because a single braille cell has six or eight points (varies according to the format; eight cells were used in this paper), which have binary values, and is expressed in a byte (8 bits). As the display contains 12×12 2D multiarray braille cells, the number of braille cells available in total is 144; therefore, to efficiently express the data on each braille cell, a transmission unit of a byte array is used. In this architecture, the visually impaired people can read the translated multimedia content and send feedback by using the keyboard recursively, as shown in Figure 14.

### 4.4. Extraction and Translation Method for 2D Multiarray Braille Display

For the implementation, this study partially used open source softwares [26,27] to extract the content of the eBook. Because they are not perfectly implemented for parsing media data in the DAISY eBooks, additional implemenation was conducted in this study; (i) a compatibility correction with the braille OS and (ii) the extraction of media content by parsing each standard. In DAISY and EPUB, the text, audio, picture, photograph, and book information can be extracted for each standard based on the markup language using tags [57].

In the relatively simple DAISY v2.02, there exists a navigation control center document and the synchronized multimedia integration language (SMIL) file corresponding to each chapter or section; moreover, the text, image, and audio contents are tagged in the SMIL file [58]. DAISY v3.0 has a less complex structure that can support more image and audio files, which was achieved by enhancing the navigation function one level higher by using the navigation control for the extensible markup language (XML) file, which is referred to as NCX, and strengthening the multimedia functions [59]. Table 3 lists the differences between DAISY v2.02 and v3.0 standards. The structure of DAISY v3.0 is similar to the EPUB 2.0 and 3.0 standards [35]. As mentioned previously, the parsing methodology varies according to the standards; moreover, the references of media file tags are also completely different. Therefore, the eBook reader also varies according to the standard so that we put together all of standards in one eBook reader application. In addition, DAISY v2.02 has no bookmark tool for the disabled. Therefore, this study implements the bookmark feature using .bmk files, which involve metadata, such as page index and chapter number of MP3 files. In addition, for audio or image content, an audio player and image viewer corresponding to the eBooks were implemented to provide visual/audio content, separately.

Conversely, DAISY v3.0 does not utilize the NCX for the implementation of the application and extracts content accurately by using the tag-parsing method, which searches at a level directly below the NCX file. The media data parsing, which uses a tagging-based method, is implemented using the follow steps. (i) A validity test of the XML file is required to process the DAISY v3.0 eBook using conditional statements. (ii) The level of the XML document of the book is extracted by searching with depth attributes. (iii) When an author creates a DAISY v3.0 book, the eBook file is composed by classifying parts, chapters, and sections using level tags. Therefore, every tag is collected and then, the chapter list and all the contents of the book are extracted. As this method outputs an XML document without going through the navigator, the computation speed is much faster while the compatibility in this study is maintained. Because the method using DAISY v3.0 open source does not perform the optimization properly, a few seconds are required to perform the braille translation. This study resolved this problem by direct tagging without using a NCX file when the current activity is moved to the next chapter activity on the eBook reader application. Therefore, the outputs of the text content are displayed in less than 1 s, which is relatively much faster.

Using epublib, an open library that supports EPUB 2.0 and 3.0, a book created using the EPUB standard can be extracted on the Android OS. Epublib is designed to facilitate metadata extraction using several tags [60]. Through the epublib library that is converted into the Android standard, parsing is performed on the braille OS by following the usual EPUB parsing method using the NCX [61]. In contrast to the open source of DAISY v3.0, the method using epublib can extract the entire content of the EPUB based on the hyper-text markup language (HTML) document without any compatibility problem. Furthermore, because it has been developed over a long time, this open source has been well optimized and debugged. Epublib extracts the contents of the eBook to a HTML document. EPUB and DAISY are based on the style of tag-based documents. Therefore, for extracting text content from books of these formats, tag tracking and tag-based content extraction is required. By using jsoup open library, the tags of corresponding content are parsed, then all the contents are extracted [62]. The extracted content are then divided into text, audio, and image contents, which are allocated to the corresponding player or viewer.

Additionally, in Algorithm 1, the text content of eBooks of different standards are distinguished sequentially. The text content that are organized by chapter are converted into braille data as follows. (i) The carriage return characters and spaces in the text are deleted, and the words are inputted in the byte array. (ii) The divided words are converted into braille. The translated words are stored with spacing in the braille buffer page, and then data are accumulated per word. When the converted data exceed the size of the braille display page size, the buffer page flushes the accumulated braille data to a braille page. Finally, if the book is completely translated to braille, the finished braille translation data are saved and outputted on the braille display. In this algorithm, row and col refer to the horizontal and vertical cell sizes of the braille display; further, words are distinguished and added to each row of the braille array. Through row and col, the maximum amount of text that can be shown on the braille display is presented. Then, the braille content of the buffer is saved, and all the variables used for allocating a page are initialized to prepare for the subsequent braille page. Even if the braille display size or source language of the original text is changed, the braille array is assigned to fit the cells in accordance with the byte array by assigning the variables through this algorithm. Through this algorithm, the text content of the book are divided into words and converted to rows×cols braille cells.

**Algorithm 1:** Text to braille conversion and text/braille page division for text content of an eBook **Input**: Text content of an eBook **Output**: braille eBook and highlighted textbook 
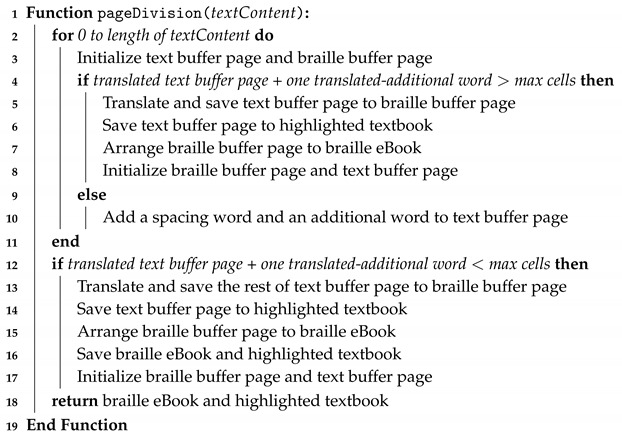


### 4.5. Braille Image Translation Based on 2D Multiarray Braille Display

**Figure 15:**
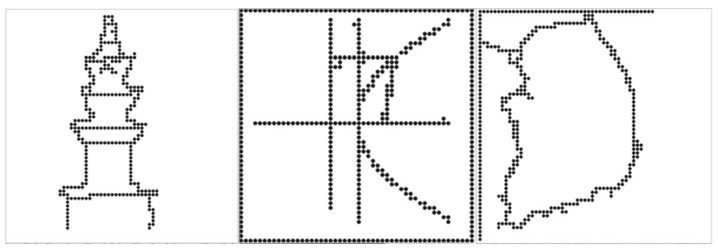
Implementation of braille figure within 50×50 multiarray braille display [33]. Converted figure on the left: tower; center: graph; right: map.

This section presents a braille figure conversion system, which was developed by InE Lab and Professor Cho at Gachon University, who conducted this joint research and has presented novel methodologies for automatic text and graphics translation to braille. Their methodology was used for figure conversion [33,63,64]. Generally, the experts on braille graphics consider three factors when constructing braille graphics: image complexity classification, tactile translation of low- or high-complexity images, and image segmentation. For a complex classification, images are considered as complex, excluding simple graphic images, such as graphs and tables. When comparing the gray-level histograms of simple and complex images, the differences are apparent. Because visually impaired people usually touch the braille display from the top-left side to the bottom-right side sequentially, it is difficult for them to understand high-complexity images immediately, even though such images are presented significantly well. In addition, visually impaired people cannot perceive other image features, such as color and texture, by touching braille cells. Therefore, the edges of the central object (mostly region of interest (ROI)) were extracted and presented on the braille device, as illustrated in Figure 15.

The central object calculates the color differences in an image and labels them for extraction. It is assumed that the central object is in the center of the image, and its objectives are to: (i) simplify color values by quantization and (ii) obtain color similarities and extract color values by separating each color in the image. Generally, based on photography and triangulation, the largest area within these labeled areas is designated as central label.

The edges of the central object area were modified to binary data and the border of the binary figure was extracted. However, according to the survey listed in Table 4, tactile graphics must be expressed simply [33,63,64]. Otherwise, the information might not be recognized accurately by the users. In the binary image, there are certain irregular sharp lines in the contours. Cell lines on the edges should be simplified to avoid recognition problems. Therefore, to remove these noises, expansion, and segmentation were conducted on the binarized image to create a simplified contour image. Then, corner points were extracted using the Shi–Tomasi algorithm [65]. Consequently, unnecessary corner points were removed to simplify the contour image. Finally, this simplification method increased the recognition rate of the central object by visually impaired people.

## 5. Implementation and Results

### 5.1. Haptic Telepresence System

**Figure 16:**
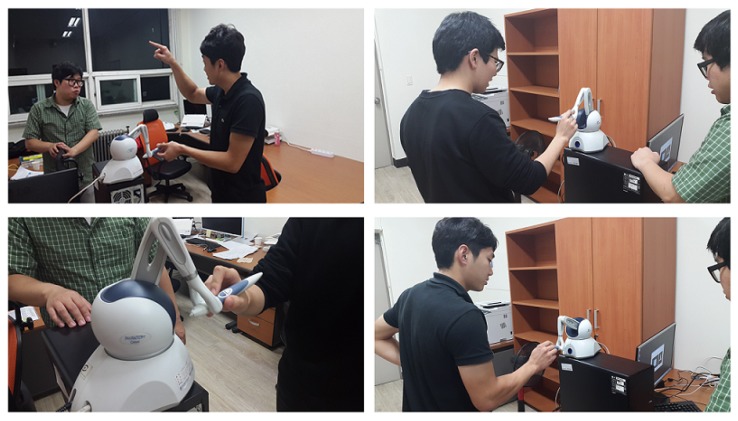
Subjects were trained on the utilization of haptic device. Haptic recognition rate had a very close relationship with the time of use.

In this study, an experiment was performed for the method of delivering objects tactually to visually impaired people by using a haptic device via Kinect v2 SDK (C# Language) and the PHANTOM Omni haptic device manufactured by SensAble Technologies. A laptop was used to implement the client role of the haptic device and a desktop was also used for the server and GUI expression. Visual studio and C++ were used for this implementation. However, in this haptic research, although the research team invited certain visually impaired people for participating in this haptic telepresence test, they were unwilling owing to various reasons, such as their schedule, their lack of interest, or their apprehensions regarding the study. Thus, to investigate the practical applicability of the haptic system introduced in this study, a test was conducted with 10 non-visually impaired participants, who were blindfolded. In general, because of the lack of certain developed abilities (i.e., recognition through touch), it was expected that the blindfolded participants would show lower haptic recognition rate than visually impaired people. Figure 16 shows our experiment.

**Figure 17:**
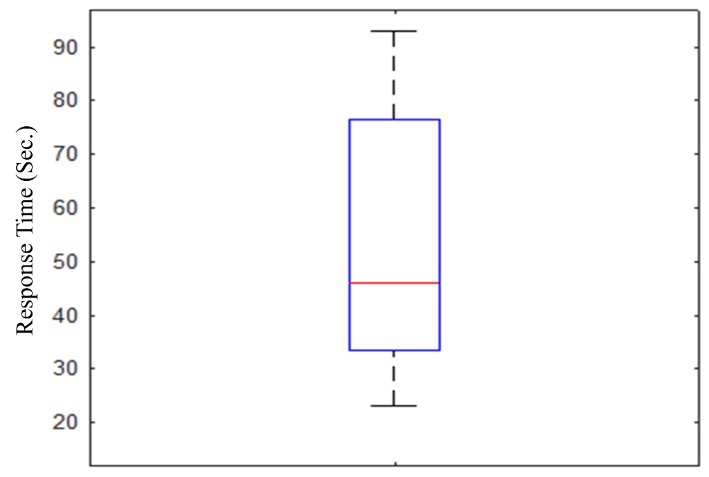
Response time of the proposed real-time 3D haptic telepresence system for 10 blindfolded participants (non-visually impaired people). The length of the box represents the 25th to 75th interquartile range. The interior red line represents the median. Vertical lines issuing from the box extend to the minimum and maximum values of the analysis variable [36].

Prior to the experiment, the experimental subjects were trained on how to use the haptic device and how an object was projected. The researchers provided random 3D shapes to subjects. Subsequently, the recognition rate, recognition time, 3D view reconstruction time, and the feedback of the subjects regarding this experiment were analyzed. The experiment was conducted as described: (i) a set of solid figures [(a) sphere, (b) cone, (c) cylinder, (d) pyramid, and (e) cube, were provided to 10 testers; (ii) the director provided one of the sets to testers randomly; (iii) the testers chose a randomly presented solid figure through tactual cues from the haptic device. Consequently, the reconstruction time was less than 1 s. It varied according to the complexity or size of the shapes. As shown in Figure 17, the experimental result shows that certain testers recognized the objects quickly, in approximately 20–30 s, while certain subjects required over 90 s. On average, the experiment subjects required approximately 35–70 s to recognize an object. This result is similar to the experimental results presented by Park et al. [10], because visually impaired people and non-visually impaired people have different levels of understanding regarding haptic devices, as was used in this experiment; consequently, a large recognition gap is recognized. If the participants were well-trained, the test result could be enhanced.

### 5.2. Braille eBook Reader Application Based On 2D Multiarray Braille Display

This study also attempted to develop a 2D braille display that was more practical and intuitive compared to haptic devices. This research made an effort to eliminate the existing disadvantages and provide convenient use while maintaining the superior features of the most popular braille device. It focused on the interaction between visually impaired people and their assistants or guardians as well, to enable smooth mirroring. Further, the information shared with the smartphone is used by non-visually impaired people. In addition, it also presents the result of the braille figure conversion system on a braille pad simulator.

**Figure 18:**
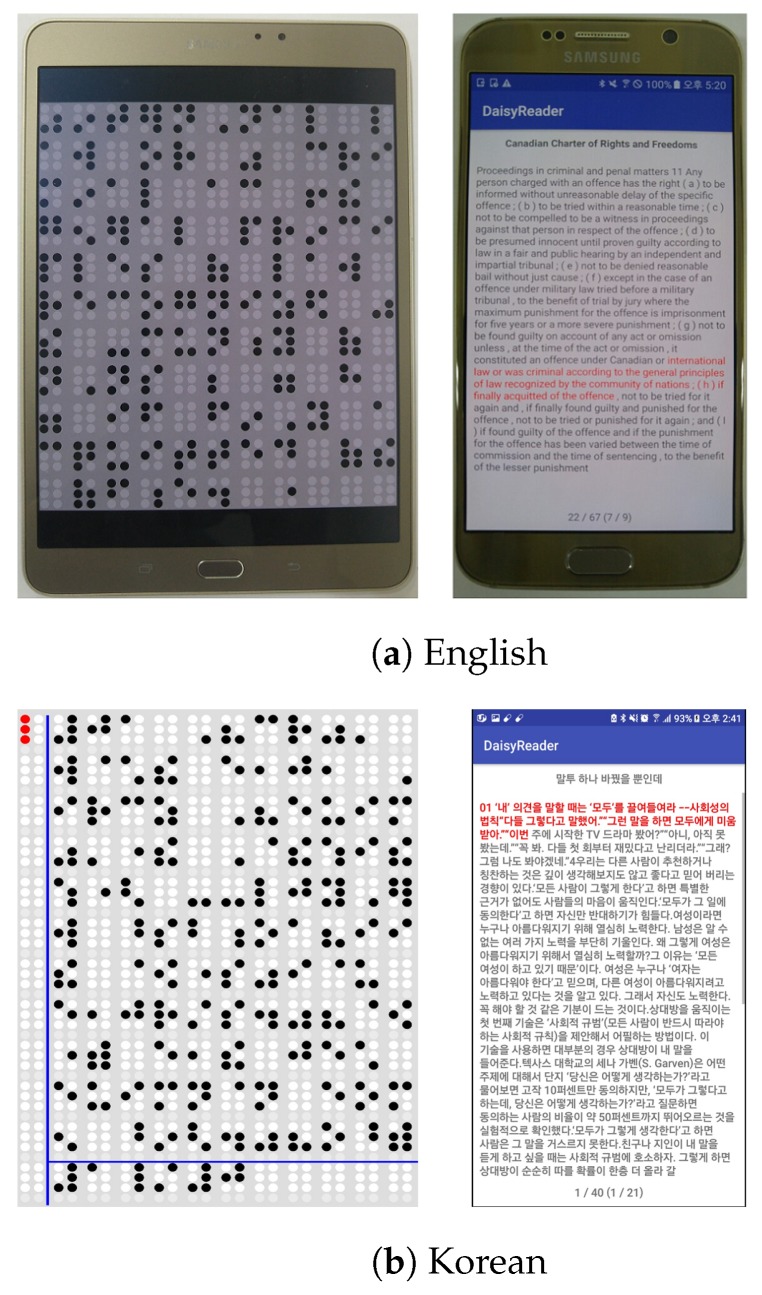
Text player with digital accessible information system (DAISY) v2.02 (English), DAISY v3.0 (Korean) eBook [37].

In this study, the experiment was performed by using a combination of a tablet and smartphone, which replaced the braille display device, as shown in Figure 18. The tablets used in this study were Samsung Galaxy Tab S and Galaxy Tab S2 8.0; moreover, Samsung Galaxy S7 Edge, S6, and Note 5, and LG G4 smartphones were used. Their OS was Android 6.0 Marshmallow. The development was conducted using the lowest SDK version of 19 for the Android application programming interface. For the Android SDK, Android Studio 2.0 and 3.0 were used, and the experiment was conducted on a laptop computer, which had Intel i3 CPU, 4 GB RAM, and solid-state drive of 500 MB/sec read/write speed. The sample files used for DAISY books were sample books provided by the DAISY Consortium and the EPUB sample files were provided by Pressbooks [66,67]. Figure 18 shows that a tablet and a smartphone were connected by Bluetooth communication, and text and braille were expressed while exchanging data through the braille OS. Figure 18b shows the braille content that a user is reading on the tablet, which was a text of DAISY v2.02 translated to braille. This illustrates an actual execution on the tablet and smartphone; moreover, the tablet screen was modified to show only the content of the multiarray braille display.

**Figure 19:**
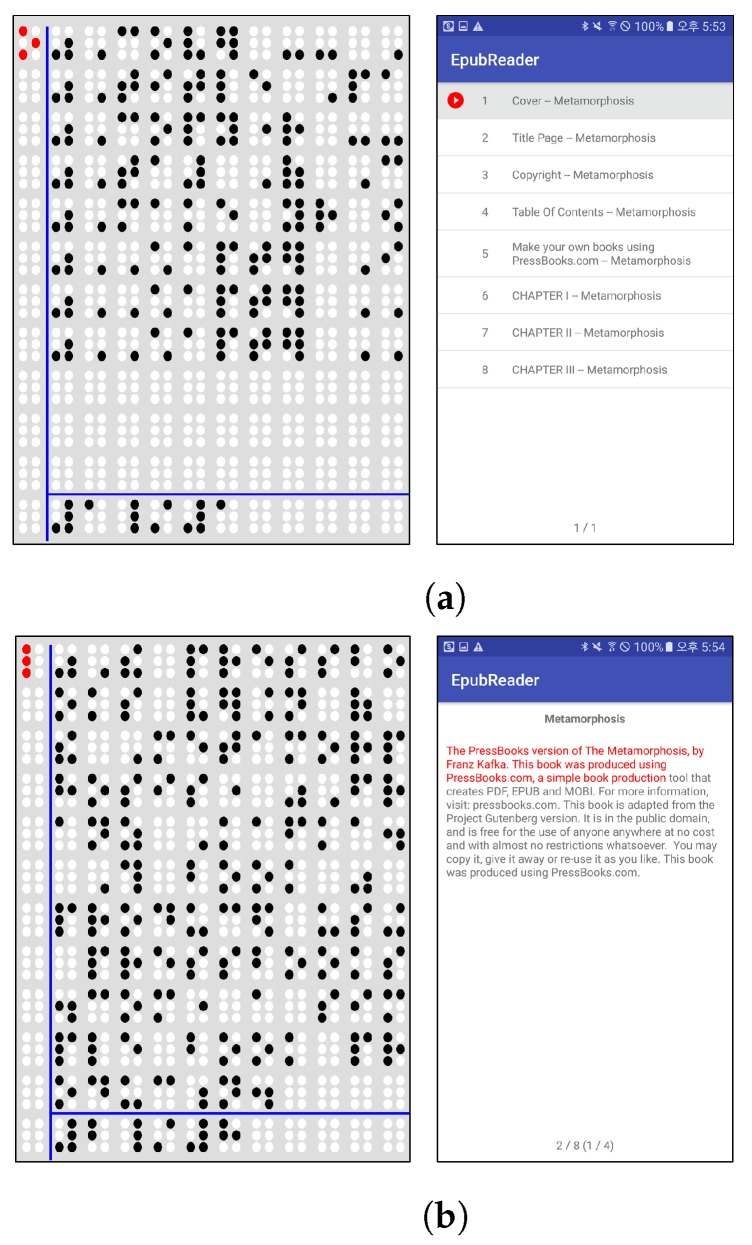
Text player with sample eBook of EPUB standard [37]. (**a**) The chapter list of electronic publication (EPUB) [37]; (**b**) The text player of EPUB.

**Figure 20:**
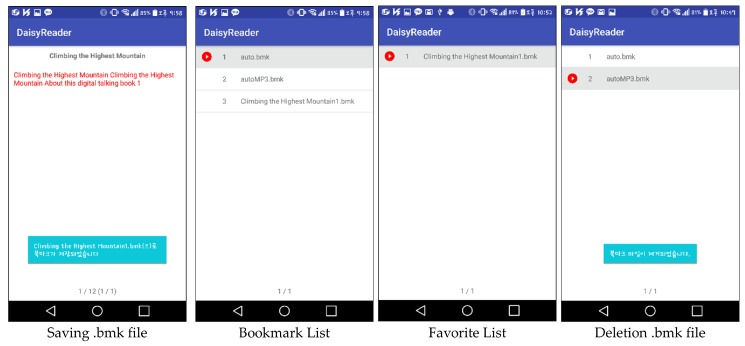
Bookmark and favorites placeholder in the braille eBook reader application.

Figure 19 shows examples using an eBook of the EPUB standard. Figure 19b shows all the chapters listed in a sample eBook is normally outputted. In addition, Figure 19b also illustrates that the text content of an eBook is normally outputted through the text player. Figure 20 shows the implemented bookmark page. DAISY v2.02 and EPUB 2.0 have no bookmark feature; however, DAISY v3.0 and EPUB 3.0 have this feature. This study provides the bookmark feature for both DAISY v2.02 and EPUB 2.0 standards.

**Figure 21:**
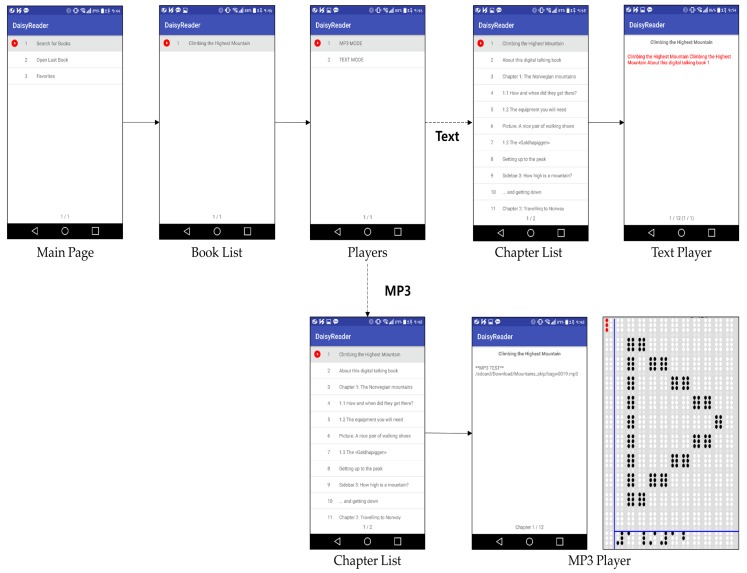
Sample progress in an eBook reader application [37].

**Figure 22:**
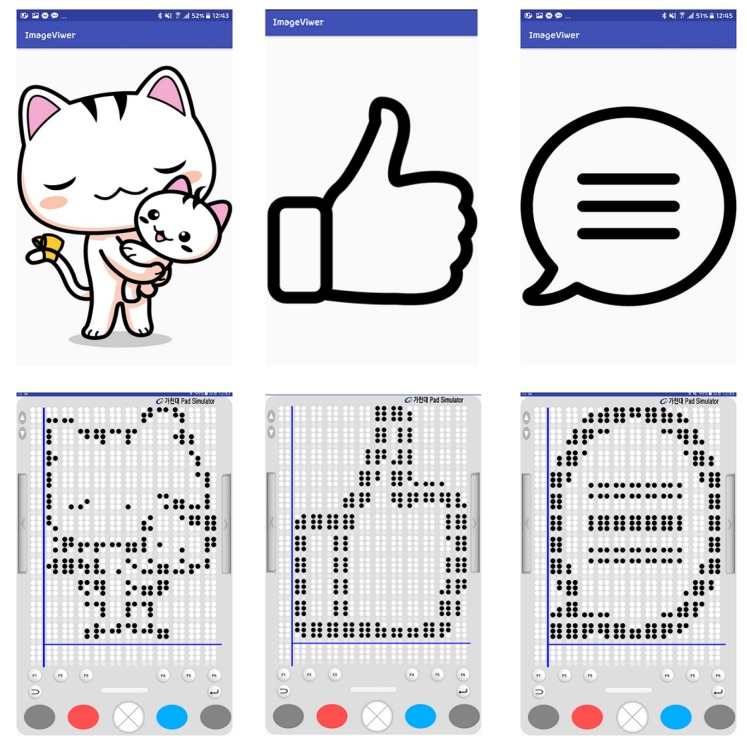
Implementation of figure conversion on a 12×12 braille pad simulator. Converted figure on the left: cat character [71]; center: like logo [72]; right: chat logo [73]. This conversion uses six digits of a braille cell per braille figure.

Figure 21 shows the sequence of processes performed when visually impaired people are reading a book with a smart phone example. In the main page, a search for books can be performed, the last book can be opened, or a favorite bookmark can be selected. When the opened last book or a favorite book is selected, the text player is executed immediately. When “search for books” is selected, the book list is opened, and this displays the list of books in the smartphone storage. Moreover, if the searched book contains music, either the audio mode or the text mode is selected, and the selected mode is executed. In this study, the modes are executed based on chapters (usually audio files are divided according to chapters). According to the selected part of the book, the book content is played. Park and Jung et al. [33,63,64,68,69,70], who collaborated with our team, conducted research pertaining to the processing of images in books, and developed a method where when a user presses a certain key in the image viewer, the book image list is displayed. In this study, if a tag of an image file is attached to a chapter that a user is currently reading, it can be tagged and extracted; thus, an image file list is created. When the user enters the image viewer by using the list, the image-oriented objects are selected and expressed, as shown in Figure 22.

**Figure 23:**
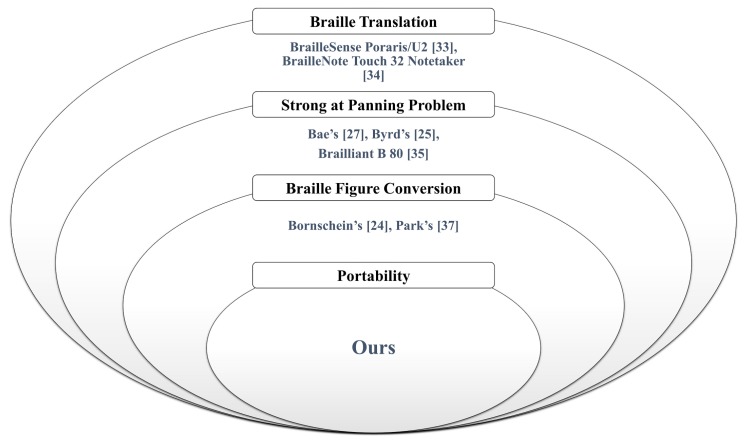
Cumulative Ben diagram based on Table 5. There are reference numbers on the diagrams, and they are classified by features. eBook standards and OS features are excluded.

Table 5 and Figure 23 present the comparison of related work in this area. Certain research focused on visually impaired people and/or people having reading disabilities. The DAISY standard, especially for people with reading disabilities, was used in most studies. Excluding EPUB, DAISY is an essential eBook standard for visually impaired people. In addition, we realized that most studies tended to develop software based on Android OS for mobility. Most Windows OSs, except Windows CE 6.0, are used for operating desktop computers. Further, some applications could not be implemented on a braille display; therefore, we could not compare the panning problem. Byrd’s method and Blitab have no information regarding DAISY and EPUB. Specifically, Byrd’s implementation uses the Windows OS, and it supports nonvisual desktop access (NVDA), developed by NV Access. NVDA allows blind and visually impaired people to access and interact with the Windows OS and many third-party applications [74]. The study by Bornschein et al. [20] compared the methods for displaying braille figures, which contributed to the education of visually impaired people; however, they concluded that the BrailleDis device was not popular as it was a tethered display; consequently, users may have felt uncomfortable while using it. Unfortunately, the proposed braille display device has not been fully developed yet. Nevertheless, to reduce the issues with the currently marketed braille displays, we resolved the limitations of the panning problem and braille figure support.

## 6. Limitation and Discussion

### 6.1. Haptic Telepresence

**Figure 24:**
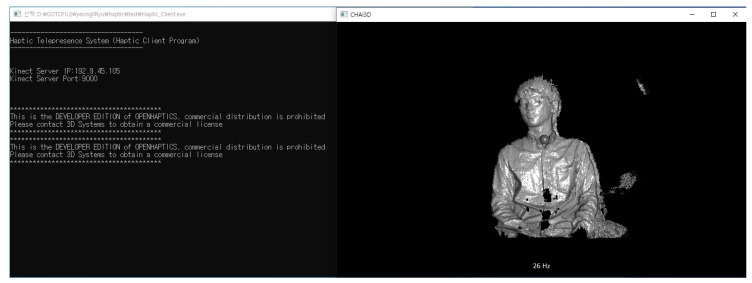
Human recognition through proposed haptic telepresence (client side).

Unfortunately, it was observed that subjects felt uncomfortable while using the 3D haptic device. They reported that it was confusing to classify similar shapes (e.g., cone and pyramid). Although the recognition rate by subjects was not low, it took longer than the anticipated time. The large differences were due to the different skill levels of individuals as well as the different levels of understanding of instructions. This research was a pre-study with non-impaired persons because recruiting the visually impaired people for this specific project was difficult. When easy-to-understand objects were expressed by subsampling of figures or long shapes, subjects demonstrated no problems in recognizing them. However, when the given shape was of a small object or was a complex shape (i.e., human-like depth map, as shown in Figure 24, it was more difficult for the subjects to recognize the shapes using the haptic device with depth maps. Although this method transfers the point cloud video in real-time without issues, when a video is produced with an IR sensor, the resolution tends to be lower. Because of these reasons, limitations were revealed while expressing and recognizing a small or complex object. Moreover, the haptic device is expensive; further, because it is large and heavy, it is difficult to carry. This implies that new technology is required to deliver more detailed information to express an object precisely and tactually and to improve mobility. Because of these problems, this research changed its direction to 2D multiarray braille display.

**Figure 25:**
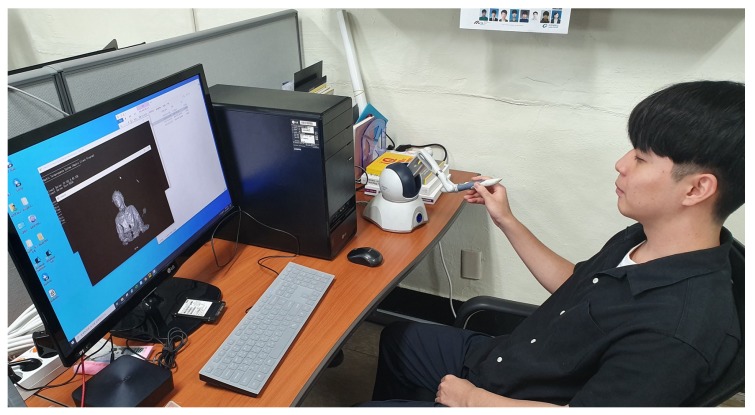
Subject is testing the recognition of a human using the proposed haptic telepresence system.

Additionally, as shown in Figure 25, the original objective of this research was the ”haptic telepresence television for visually impaired people.” It can enable visually impaired people to meet others remotely using certain applications, such as Skype (from Microsoft), or observe the hand-held screen of a TV. However, as the research progresses, this research team was skeptical about the effectiveness of the haptic device, as it had demonstrated unclear results regarding practical haptic telepresence. This implied that a novel technology was required to capture the object and deliver more details while expressing an object tactually. Currently, it is difficult to express tiny or complex objects using a haptic device.

### 6.2. Braille eBook Reader Application

**Figure 26:**
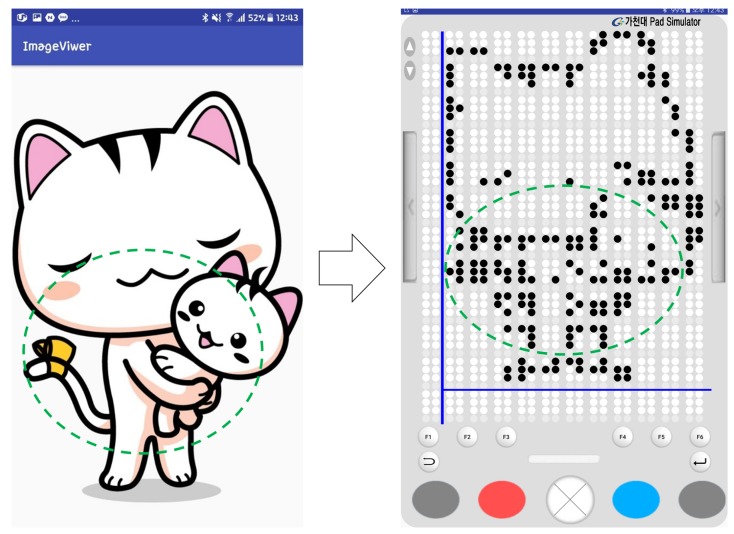
Limitation of braille figure conversion. Green circles indicate the same position between the original and the converted braille image. The braille figure shows unclear boundaries in 12×12 braille cells.

The braille display device demonstrates a superior performance than the haptic device while delivering media information. However, several components have to be implemented without helpful information while designing the braille display device. Unlike other software, the software required for this device has not been developed well. The research on open source softwares or software/applications related to DAISY, braille refreshable format, and EPUB standards are considerably scarce. In addition, it is still difficult to let visually impaired people touch and feels images or videos through the braille pad. In this study, a video was expressed with only the cover image of the video. Further, images expressed on the braille pad simulator were more unclear than the images appearing in teaching materials or books. Originally, the main objective of this study was to provide ways to educate the visually impaired people. A new format of tactile cells is required to express photographs and pictures that contain many objects on a small braille display. As shown in Figure 26, the braille figure looks like a raster image. It has several more artifacts than the original vector image, because the number of pixel and braille dots are significantly different. The original figures are depicted imperfectly within limited number of dots in a braille display.

Specifically, this study was unable to prove the developed applications in less developed prototypes. Although this study considered a method for expressing a video on the braille pad as well, this requires further research on the reaction speed of actuators, the power consumption, design, etc.. While considering the braille hardware, there are several challenges that have to be solved in terms of software, such as video sampling, filtering, and streaming. Hence, this study attempted to use a method for expressing a typical cover image of the video. This also was difficult to express properly because cover images have to be expressed through accurate filtering and conversion. Therefore, more studies are required for adequate expression of images and videos on a braille display or other tactile display. Assuming that advances will be made in hardware using superior technologies, the final objective of this study is to output videos on the multiarray braille device. The authors hope that liquid- and solid-like display devices will be studied for a novel tactile display.

## 7. Conclusions

This paper explains two main systems for visually impaired people: (i) converting a video and delivering tactile information remotely by using a 3D haptic device, and (ii) delivering braille data and multimedia content by using a 2D multiarray braille display. The first study enables visually impaired people to feel 2D and 3D object contour that cannot usually be felt remotely. To enhancing the object recognition performance, a new 2D braille system is designed in the second study. This study overcomes the limitations of traditional one-dimensional braille devices. In the implemented system, the text content is expressed within multiple lines, and it enables the visually impaired people to feel eBooks more efficiently. Moreover, a study provides the way to express images on the braille display by extracting central-labeled object.

Implemented 2D multiarray braille system displays massive textual content and other media content simultaneously. It increases the accessibility of visually impaired people to multimedia information and affects the 3D and 2D haptic information delivery for the education of visually impaired people. This research is work in progress and has the purpose to explore the possibilities to implement new devices for the benefit of the visually impaired.

## Figures and Tables

**Table 1:** Global numbers affected and the prevalence of vision impairment (approximate values) according to age and sex in 2015 (data are expressed in % (80% uncertainty interval) or number (80% uncertainty interval)).

Sex and Age (Years)	World Population (Millions)	Blind		Moderate and Severe Vision Impairment		Mild Vision Impairment	
**Men**		**Prevalence (%)**	**Number (Millions)**	**Prevalence (%)**	**Number (Millions)**	**Prevalence (%)**	**Number (Millions)**
**Over 70**	169	4.55	7.72	20.33	34.53	14.05	23.85
**50–69**	613	0.93	5.69	6.78	41.57	6.46	39.65
**0–49**	2920	0.08	2.46	0.74	21.66	0.81	23.61
**Women**							
**Over 70**	222	4.97	11.06	21.87	48.71	14.57	32.45
**50–69**	634	1.03	6.52	7.48	47.46	6.99	44.35
**0–49**	2780	0.09	2.56	0.82	22.68	0.89	24.64

**Table 2:** Comparison of popular mobile braille displays.

Product	Special Feature	Media Support	Braille Cells	Cost (USD)	OS	Release (Year)
Blitab [28]	Displaying braille image	Image and audio	14×23	Unknown	Android	Unknown
BrailleSense Polaris [29]	Office and school-friendly	Audio-only	32	5795.00	Android	2017
BrailleSense U2 [29]	Office and school-friendly	Audio-only	32	5595.00	Windows CE 6.0	2012
BrailleNote Touch 32 braille Notetaker [30]	Smart touchscreen keyboard	Audio-only	40	5495.00	Android	2016
Brailliant B 80 braille display (new gen.) [31]	Compatibility with other devices	Text-only	80	7985.00	Mac/iOS/Windows	2011

**Table 3:** Comparison of digital accessible information system v2.02 and v3.0 standard (media files involve text, image, and audio file).

Standard	DAISY v2.02	DAISY v3.0
Document	based-on HTML	based-on XML
Configuration	Media files, SMIL, and NCC	Media files, SMIL, NCX, XML, and OPF
Reference file	NCC file	OPF file
Metadata Tag	<title>, <meta>	<metadata>
Context Tag	<h>	<level1>

**Table 4:** Survey results on tactile graphic recognition characteristics of the visually impaired (15 participants were completely blind, 10 participants had low vision) [33].

Item	Recognition Characteristics
Image details	Expressing excessive detail can cause confusion in determining the direction and intersection of image outlines. Therefore, the outline in both low-and high-complexity images should be expressed as simply as possible to increase the information recognition capabilities.
High-complexity image with a central object	For an image containing a primary object, the background and surrounding data should be removed and only the outline of the primary object should be provided to increase recognition capabilities.
High-complexity image without a central object	For an image without a primary object, such as a landscape, translating the outline does not usually enable the visually impaired to recognize the essential information.

**Table 5:** Comparison of assistive applications and devices in this study (DAISY: v2.02 and v3.0, EPUB: 2.0 and 3.0); the ∘ means ‘support’, and the × means ‘not support’.

Research	eBook Support (DAISY/EPUB)	Portability	Braille Translation	Braille Figure Conversion	Panning Problem	OS
**Application**						
Bae’s [23]	DAISY	Weak	∘	×	Strong	Windows
Kim’s [24,25]	DAISY	Strong	×	×	-	Android
Bornschein’s [21]	Unknown	Weak	∘	∘	Strong	Windows
Bornschein’s [20]	Unknown	Weak	∘	∘	Strong	Windows
Goncu’s [19]	EPUB	Strong	×	×	-	iOS
Harty’s [26]	DAISY(v2.02)	Strong	×	×	-	Android
Mahule’s [27]	DAISY(v3.0)	Strong	×	×	-	Android
**Braille device**						
Byrd’s [22]	Unknown	Strong	∘	×	Strong	Windows (NVDA)
Park’s [33]	Both	Weak	∘	∘	Strong	Windows
BrailleSense Polaris [29]	Both	Strong	∘	×	Weak	Android
BrailleSense U2 [29]	Both	Strong	∘	×	Weak	Windows CE 6.0
BrailleNote Touch 32 Braille Notetaker [30]	Both	Strong	∘	×	Weak	Android
Brailliant B 80 (new gen.) [31]	Both	Strong	∘	×	Strong	-
Ours	Both	Strong	∘	∘	Strong	Android

## Abbreviations

The following abbreviations are used in this manuscript:

DAISY	Digital accessible information system
HEVC	High efficiency video coding
SHVC	Scalable high efficiency video coding
LED	Light emitting diode
IR	Infrared
EPUB	Electronic publication
TTS	Text-to-speech
NCC	Navigation control center
NCX	Navigation control center for extensible markup language
XML	Extensible markup language
SMIL	Synchronized multimedia integration language
HTML	Hyper text markup language
SDK	Software development kit
